# Balancing tradition and innovation: rethinking the dichotomy in anatomy teaching

**DOI:** 10.3325/cmj.2025.66.62

**Published:** 2025-02

**Authors:** Marina Čavka, Zdravko Petanjek, Maura Zanze, Beader, Thomas Mathew, Cecilia Brassett, András Dávid Nagy, Ana Hladnik

**Affiliations:** 1 Department of Anatomy and Clinical Anatomy, University of Zagreb School of Medicine, Zagreb, Croatia; 2 University of Cambridge, Human Anatomy Centre, Department of Physiology, Development and Neuroscience, Cambridge, United Kingdom; 3 Department of Anatomy, University of Pécs Medical School, Pécs, Hungary

## Abstract

Medical education has undergone a significant reform in response to evolving healthcare demands, technological advancements, and emerging research in biomedical and education sciences. The teaching methods in anatomy, a core component of medical education, have notably changed, with traditional cadaveric dissection being increasingly replaced by digital and hybrid alternatives. Despite research indicating no significant difference in short-term knowledge retention between students who engaged in cadaveric dissection and those engaging in alternative methods, dissection uniquely fosters professionalism, empathy, and ethical awareness – traits essential for holistic medical education. This review critically examines the dichotomy between traditional and innovative teaching methods in anatomy education, questioning the assumption that traditional methods hinder progress in modern healthcare. The findings suggest that changes in medical education are primarily influenced by organizational issues, which frequently results in an incomplete implementation of innovative teaching approaches. The inconsistent application of innovative teaching methods makes it difficult to assess their effectiveness and compare them with traditional methods. Reliable data on their long-term impact can only be generated by randomized controlled trials and longitudinal studies. In the meantime, we need to ensure that current medical students receive high-quality education by incorporating best practices from diverse teaching methods based on valuable insights from experienced educators and current students' learning preferences.

Over the past century, we have witnessed changes in health policy priorities, an increased need for healthcare professionals, along with unprecedented technological innovation and emerging research in biomedical and education sciences. All of this has led to a constantly evolving medical education (1,2). As the quality of medical education has always been important for society, questions have been raised about the effect of innovations in teaching methods and curriculum reforms on the professional development of future doctors (3,4).

Initiated through the LEANbody project (5), this review aimed to explore these questions through the perspective of anatomy — a fundamental component of medical curricula globally and a subject that has been significantly influenced by changes in course structure and teaching methods (6). Our primary research question was whether there are elements of traditional anatomy teaching that are essential and cannot be eliminated without significant loss of quality or prestige.

To explore this, we first examined the underlying drivers of the mentioned changes, which should ideally be guided by research evidence and tailored to meet specific societal needs. The World Federation for Medical Education recognizes that medical education is more shaped by societal factors than by research results. This is why its global standards for quality improvement encompass broad areas such as student support and curriculum development without mandating specific methods, thus allowing institutions to adapt the guidelines to their unique cultures, resources, and goals (7). However, increasing organizational challenges often prompt changes that can compromise educational quality. For example, the shortening of courses (8), increasing student enrollment (9), the lack of qualified anatomists, decreased funding, and limited access to cadavers (10) have all contributed to the declining role of traditional cadaveric dissection in many anatomy programs. A meta-analysis showed that students who engaged in cadaveric dissection performed equally well on knowledge-based exams as those who learnt anatomy through alternative methods such as prosections, digital media, models, or hybrid approaches (11). However, while building a strong foundation of basic knowledge is essential in preparing students for clinical practice, the ultimate goal of medical education extends beyond short-term knowledge retention and academic performance. What truly defines students’ readiness for the demands of their future careers is the development of competency across various professional domains (12). In this context, some researchers have raised concerns about the potential loss of unique benefits associated with cadaveric dissection, especially the “hidden curriculum” that fosters non-technical skills such as professionalism, empathy, and ethical awareness (13).

Therefore, the aim of this review was to critically evaluate the perspective that traditional didactic methods in medical education, particularly in anatomy teaching, are ineffective and hinder progress in meeting the challenges of global healthcare in the 21st century (14,15). This viewpoint argues that traditional methods should be replaced by innovative educational approaches to better prepare students for the complexities of modern medical practice. Through this review, we aimed to assess whether these claims are substantiated by evidence and to explore whether a balance between traditional and innovative methods might provide the most effective framework for medical education.

## The long history of anatomy: where does traditional anatomy begin and end?

As evident from the etymology of the word “anatomy,” which is derived from the Greek *anatome* meaning “to cut” or “to cut repeatedly” (16), dissection has been an integral part of anatomy teaching throughout history. Dissection typically followed theoretical instruction, offering students a hands-on, immersive experience and an opportunity to observe the organization of the human body and appreciate the texture of tissues (17). This method also allowed students to understand the relationships between anatomical structures, recognize anatomical variations, and develop the technical skills essential for their future professional practice (18).

A traditional anatomy course is typically delivered through in-person lectures, studying from anatomy textbooks, and performing hands-on cadaveric dissection in the laboratory (9). All these methods combine to enable medical students to gain a detailed and comprehensive understanding of the structure of the human body (19).

Traditional lectures help students to develop a theoretical foundation for understanding anatomy and offer the context for what they will observe in cadaveric dissection. These lectures mainly emphasize the transfer of knowledge from the lecturer to the students (20), where the lecturer describes anatomical structures and explains their functional and clinical significance (21).

Moreover, traditional, topic-based curricula primarily focus on delineating the content that educators are expected to teach (9,22,23), whereas constructive alignment has now become a crucial aspect of quality assessment. Constructive alignment was conceptualized by Biggs in 1996 (24) based on earlier work by Tyler in 1949 (25) and Shuell in 1986 (26). As a form of outcomes-based teaching and learning, it ensures that both instructional strategies and assessments are connected to the intended learning outcomes, which specify what students are expected to do with the knowledge they acquire (27).

Whereas modern curricula increasingly emphasize the effectiveness of active learning strategies that promote student-driven knowledge construction — such as flipped classrooms, problem-based learning (PBL), and team-based learning (28) — traditional teaching is often perceived as relying on passive learning. However, it is difficult to evaluate the effectiveness of different teaching methods because of the variability in implementation and differences in students' learning habits (29,30).

Many of these methods, often regarded as “innovations,” also have roots that extend further back than is commonly assumed. For instance, PBL can be traced back to the early 20th century (31). The hands-on approach in the anatomy classroom evolved around the same time to include a broader range of active-learning methods, such as peer learning and reflective discussions. With increased availability of practical materials and decreasing lecture time, these strategies encouraged students to engage directly with the material and learn from each other, often in small groups, effectively blurring the lines between traditional and modern educational practices (32).

Thus, when evaluating the effectiveness of teaching methods in anatomy education, it is essential to consider their historical evolution and the broader educational context in which they have developed. Before the mid-20th century, access to textbooks and anatomical atlases was limited. As a result, lectures were the main source of anatomical knowledge, while prosections and cadavers were the primary tools for visualizing the three-dimensional structures. The lack of technological resources required students to adopt a more proactive and self-directed approach to learning, encouraging a strong sense of initiative. Since study materials were not readily available, students often collaborated in small groups, which facilitated teamwork and peer learning. In many medical schools, students were provided with ongoing feedback by practical work assessments during classes. Passing these assessments was often a requirement for continuing with dissection.

**Figure 1:**
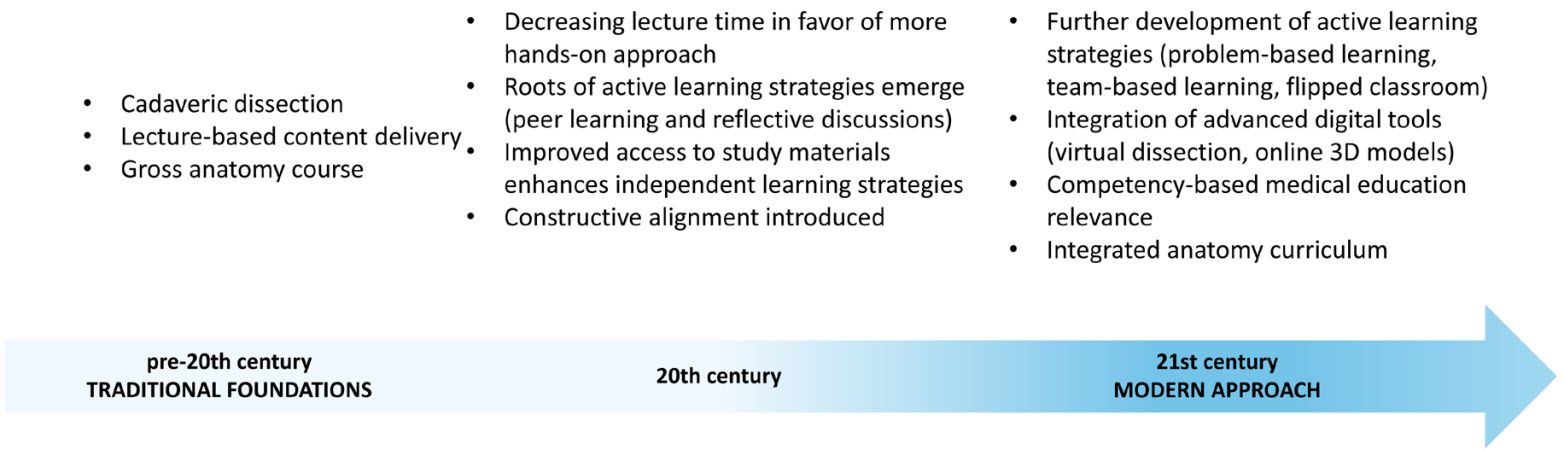
The evolution of anatomy education. The long history of anatomy education is better understood as an evolving continuum rather than a dichotomy between traditional and modern methods. Many modern approaches, such as peer and active learning, have historical roots in the 19th and early 20th centuries. The enrichment of these approaches through pedagogical and technological advancements has introduced a variety of innovative teaching methods and improved outcomes assessment. While these advancements cater to the learning preferences of current students and effectively address organizational challenges, traditional methods like cadaveric dissection continue to hold significant value.

Therefore, peer and active learning are not recent innovations; they have origins in the 19th century and have been further enriched by pedagogical and technological advancements in anatomical education (Figure 1) (33).

Interestingly, recent research has indicated that traditional lectures are adaptable and can be effectively integrated into a student-centered unit design that is both research-informed and evidence-based (34). This suggests that creating a dichotomy between traditional lectures and active learning is misguided, as it hinders a nuanced exploration of the full range of possibilities and generates bias in research agendas.

By framing the debate as traditional vs modern, we risk creating an either-or scenario that overlooks the strengths of different approaches. For instance, cadaveric dissection, a hallmark of traditional anatomy education, is often reduced to merely a method of teaching detailed structural knowledge. However, an overemphasis on memorization of structures alone diminishes its broader educational value, including the cultivation of professionalism, empathy, and ethical awareness (35). Therefore, rather than being a rigid traditional method, cadaveric dissection integrates valuable elements of active learning and contributes to the holistic development of medical students (36).

## Technological advances in anatomy education: lessons from the COVID-19 pandemic

Increasing technological advances in recent decades have led to the introduction of numerous digital tools, such as virtual cadaveric dissection, online 3D models, and videos into anatomy classrooms (9), offering alternatives to traditional cadaveric dissection and face-to-face teaching methods (37,38).

However, while digital tools provide new ways for students to engage with the material, they also bring challenges. Anatomy is inherently complex, requiring students to integrate their knowledge of systems, structures, and clinical relevance. When advanced technology is not managed appropriately, it can overwhelm students and exceed their cognitive limits, especially when they are trying to learn basic anatomy and simultaneously navigate a new tool’s interface (39). In our experience, there is also a risk of passive learning behaviors, such as rewatching video lectures or merely browsing through slides without actively engaging with the content.

Virtual cadaveric dissection tools offer interactive 3D techniques that enhance spatial understanding, provide safer and more hygienic environments, allow repeatability, and develop digital skills while saving time. As the logistics of organizing a cadaveric dissection remain a challenge (8-10), digital tools for 3D visualization of the human body may offer promising alternatives for learning anatomical structures and developing manual skills (40).

However, they may be costly, prone to technical issues, lack tactile learning experiences, and feel monotonous compared with traditional cadaver-based methods (37). In addition, many virtual reality modalities are only designed for a single user. As good teamwork is a key to the success of healthcare management, the use of virtual worlds to create scenarios that require teamwork and communication should be more widely explored (38).

Although the COVID-19 pandemic posed major challenges for educational organization, it also provided opportunities for a comparison between online and in-person classes (9,41). As the pandemic ended only recently, the available research in this area is mostly supported by evidence from students' academic performance and satisfaction rates, while the long-term effects of different teaching models on learning outcomes remain unknown. Nonetheless, important implications for the future can be drawn from the recent literature.

A systematic review showed no statistical difference in the performance of online anatomy and traditional (“face-to-face”) teaching methods. However, students reported a higher level of satisfaction with face-to-face teaching (9).

Research has found that multi-modal learning approach combining online with face-to-face educational modalities was efficient and successful (9,42). This is especially interesting with regard to the logistical challenges of organizing a course for a large number of students and the future of hybrid courses (43). Disadvantages reported by online learners, such as screen fatigue and isolation, highlight the importance of targeted interventions and strategies to improve the quality and effectiveness of online education (9,44).

Nevertheless, the vast majority of undergraduate students found anatomical dissection and practical work in general to be the most important aspect of teaching, which could not be replaced by online learning (45,46). Interestingly, current research emphasizes the importance of cadaveric dissection not only as an anatomy teaching tool but also as a means of developing professionalism of future doctors (13,47,48). While this notion had been recognized previously, it was extensively revisited during the COVID-19 pandemic, as many medical schools were forced to abandon in-person cadaveric dissection (49,50). Besides learning anatomical knowledge, direct involvement with dissection during undergraduate training allows students to practice communication and collaboration, and develop their professional identity (51,52).

Our findings (41,53,54) suggest that well-designed courses that balance technological tools with traditional practices could provide a comprehensive framework for anatomy education, fostering both technical skills and essential humanistic qualities in medical students. Students should be gradually introduced to technology while preserving hands-on learning and collaborative experiences. Such an approach fosters student engagement and reinforces learning, while minimizing the potential disadvantages of digital tools. As most schools have transitioned from a purely traditional cadaver-based curriculum to more interactive, custom-made approaches that better suit the learning preferences of new generations, more research is required to assess the best ways of integrating digital tools into the anatomy course.

## Curricular changes in anatomy education: the evolving roles of students and teachers

Traditionally, anatomy curricula have centered on gross anatomy taught during the first year of medical school, often involving cadaveric dissection and a detailed study of anatomical structures. This classical approach has long been associated with providing strong foundational knowledge, which is necessary for developing clinical skills and reasoning (3).

In a time of heightened accountability in the education profession, teachers are responsible for ensuring that all graduates are fully prepared for practice. Consequently, competency-based medical education (CBME) has entered the focus of medical education planners, outlining essential competencies for graduates and ensuring these are taught, assessed, and acquired (55).

CBME advocates favor a curriculum organized around competencies rather than extensive lists of knowledge objectives. They argue that objective-based methods often overemphasize knowledge, neglecting skills, attitudes, and the integration of knowledge necessary for medical practice (55). As a result, many schools are shortening the length of their anatomy courses, with some adopting more integrated curricula and moving away from standalone anatomy courses (56). Our experience indicates that the transition toward integrated curricula has also been influenced by a shortage of qualified educators in pre-clinical disciplines, as clinicians are often recruited to teach these subjects.

While CBME effectively addresses organizational challenges and focuses on learners achieving predefined milestones, its practical focus may inadvertently exclude content or experiences that do not directly contribute to program outcomes (55). This raises important questions as to how students should develop excellence and advanced competencies, such as becoming medical experts, communicators, health advocates, lifelong learners, managers, and scholars (2).

**Figure 2:**
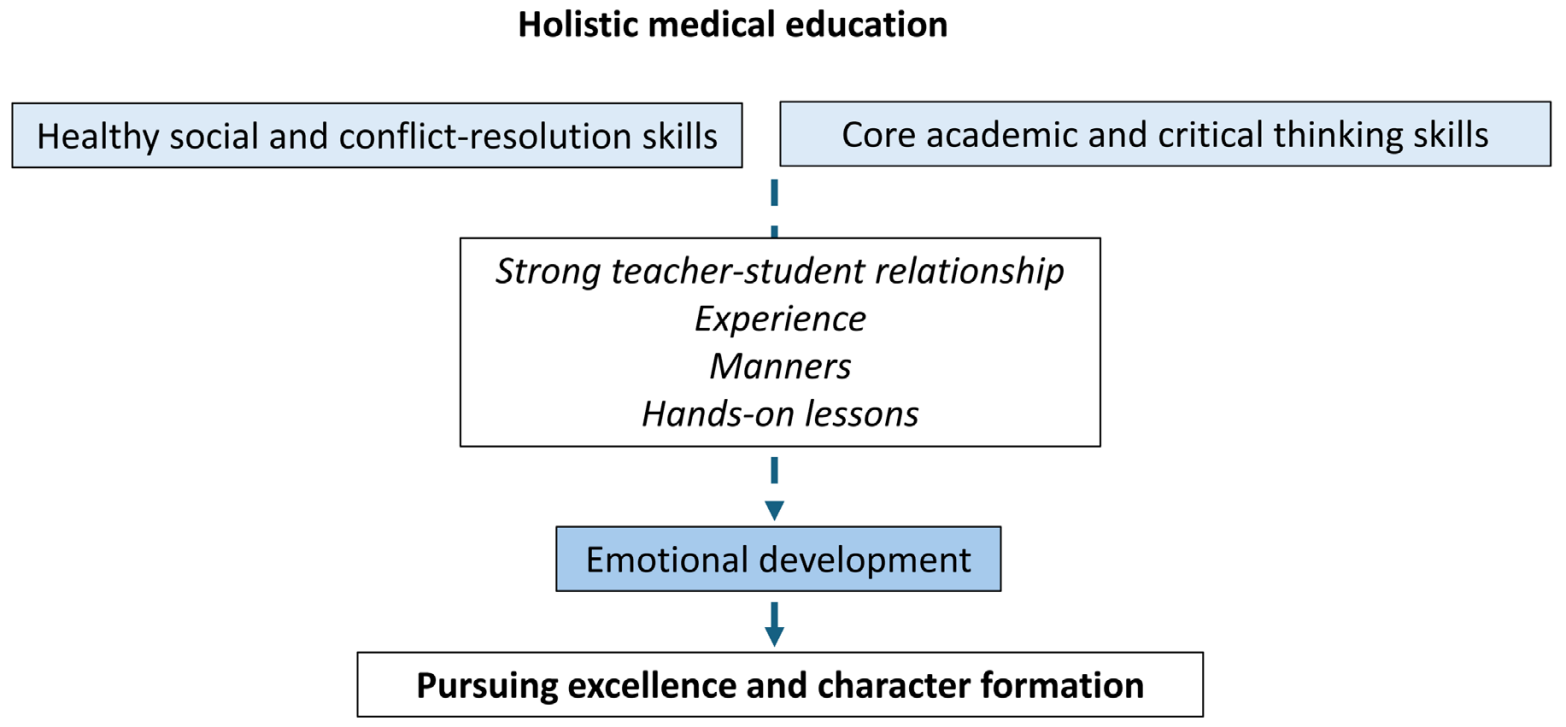
Key components of holistic medical education. This model underscores the multifaceted nature of medical education, emphasizing that it extends beyond the acquisition of foundational knowledge and practical competencies. It highlights the importance of cultivating critical thinking, emotional intelligence, and communication skills. It promotes the development of well-rounded healthcare professionals who are not only scientifically grounded and clinically skilled but also compassionate, ethically conscious, and capable of providing patient-centered care. In this way, they are better prepared to navigate the complex and evolving healthcare landscape with empathy, integrity, and a commitment to lifelong learning.

Beyond the explicit goals of anatomical education — to teach the structure and function of the human body — there are hidden objectives that may aid in developing these competencies (32). These include teaching the scientific method, addressing ethical issues, exploring topics such as death and dying, and balancing clinical detachment with empathy (13,57,58). By incorporating these broader dimensions, medical education moves beyond technical proficiency to cultivate holistic, thoughtful, and ethically grounded practitioners (Figure 2).

Historically, prominent figures in anatomy education, such as Franklin Paine Mall and Drago Perović, emphasized the role of anatomical institutes and the value of early exposure to the scientific method (32,59). The lasting effect of their mentorship on generations of leading scientists and clinicians highlights the importance of individualized guidance and personal engagement within the traditional curriculum. These figures underscored the significance of close interpersonal relationships between students and teachers, fostering both intellectual curiosity and the development of professional attitudes (60). This more individualized approach provided space for personal reflection, deeper engagement with the subject matter, and mentorship, which shaped students’ identities as future healthcare professionals.

In our experience, a demanding course such as gross anatomy could catalyze psychological and emotional maturation. It challenges individuals to step outside their comfort zones, fostering resilience, self-discipline, and critical thinking. At the same time, students should be taught coping strategies and mechanisms to improve both psychological well-being and academic achievement (61).

As cognitive neuroscience shows, adolescence is a period of significant brain development. Many functions mature into the mid-20s, which is the typical age when students enter higher education (62,63). These findings suggest that the traditional placement of anatomy education in the first year of medical training may still be relevant (21) despite the shift toward an integrated curriculum, as it introduces students to the scientific and professional aspects of medicine during a critical period of cognitive maturation. Therefore, strategically integrating anatomy education within medical curricula is essential for supporting both foundational knowledge and the development of professional and personal competencies.

Although generations of respected physicians and scientists were educated within the framework of historical practice, educational approaches should be adapted to meet the needs of Generation Z students, who have grown up in a technology-driven world and whose learning styles align with student-centered pedagogies (64,65). Still, the traditional role of teachers remains important, as students need role models while developing their professional identities and sense of belonging (66).

The hidden curriculum, which includes implicit lessons on ethical mindsets and social skills, remains vital to medical education. The transition from a teacher-centered to a student-centered approach has not eliminated the challenge of effectively conveying the hidden curriculum (67). Some authors propose that the hidden curriculum can be made more explicit in higher education when teachers align their actions with their teaching philosophy and recognize that students’ learning is shaped by their interpersonal relationships with educators (67).

Our findings suggest that early exposure to traditional gross anatomy course supports hidden objectives necessary for the professional development of future doctors. At the same time, new approaches such as CBME help graduates meet the evolving demands of modern healthcare. Ultimately, integrating traditional and modern pedagogies seems essential for cultivating professionalism, foundational knowledge, and practical competencies required in contemporary healthcare. When planning an anatomy course, educators, embracing their role as mentors who guide and shape the next generation of physicians, should recognize that students are still undergoing cognitive development (63) and differ in terms of emotional maturity, learning experiences, and preferred learning tools (65).

## Conclusion

The findings elaborated in this manuscript demonstrate a consistent aim throughout the history of medical education: to enhance the quality of anatomy teaching by evolving pedagogical approaches while addressing organizational challenges and societal needs. Distinguishing between traditional and innovative methods of teaching anatomy is challenging — and perhaps not essential. Instead, the shift toward modern methods and the ongoing evolution of medical education should be viewed as part of a continuum focused on improving teaching strategies.

While research in this field continues to evolve, each new generation of medical students requires the highest-quality education during their formative years, which underscores the need for a critical evaluation of changes in medical education. In our view, traditional didactic methods remain relevant and capable of addressing the challenges of the 21st-century healthcare system. Traditional detail-oriented anatomy teaching through cadaveric dissection continues to add value to medical education by fostering both technical expertise and humanistic qualities in future doctors. Instead of abandoning traditional methods, integrating them with technological tools and emerging pedagogical approaches may offer a balanced, effective framework for high-quality professional development.
